# Supplementary figures and images for: Short-term treatment with a peroxisome proliferator-activated receptor α agonist influences plasma one-carbon metabolites and B-vitamin status in rats
Source: PLoS One. 2019 Dec 5;14(12):e0226069. doi: 10.1371/journal.pone.0226069 (PMC6894826; doi:10.1371/journal.pone.0226069)

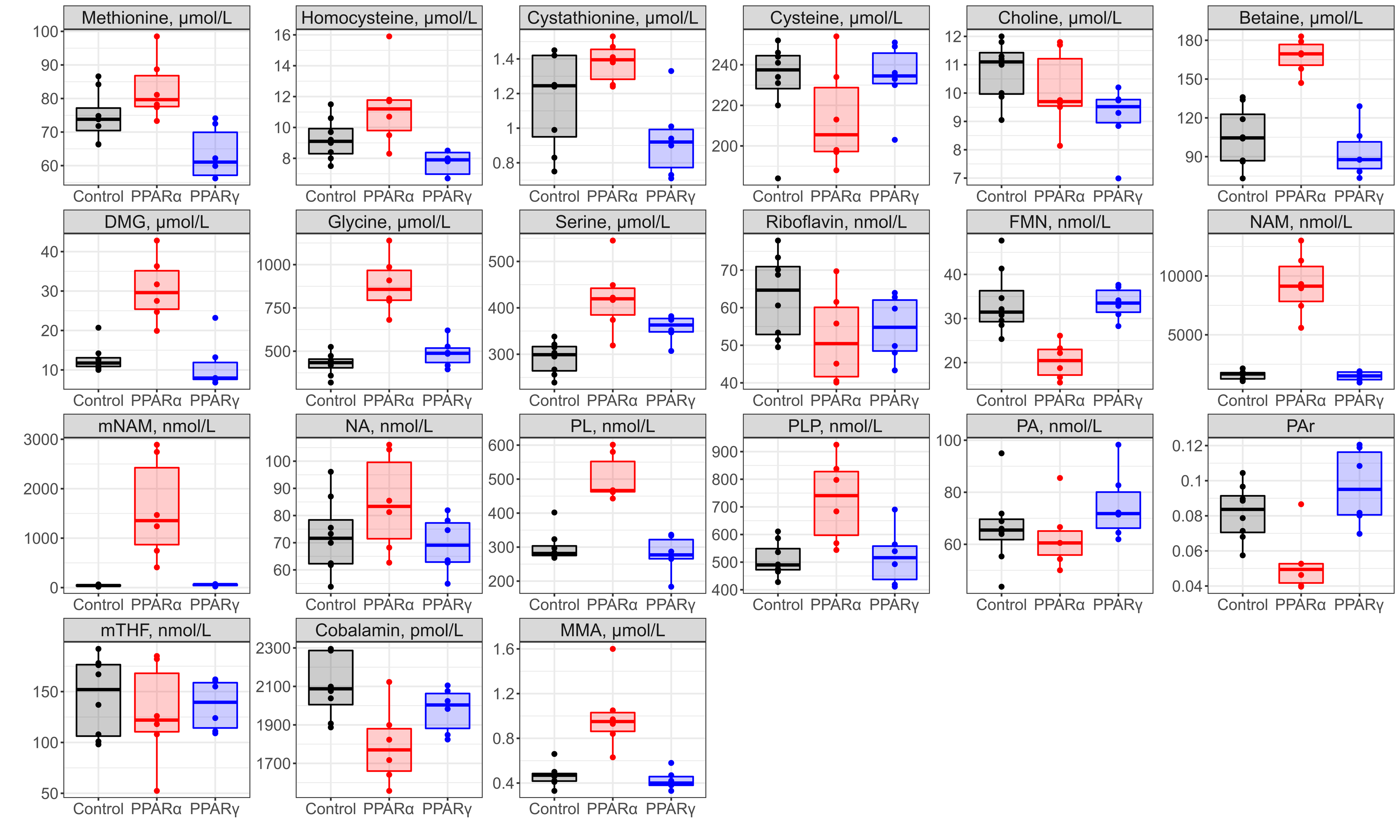

Supplement: S1 Fig — Black dots represents the control group, red dots represents the PPARα group and blue dots represents the PPARγ group. DMG, dimethylglycine; FMN, Flavin mononucleotide; MMA, methylmalonic acid; mNAM, methylnicotinamide; mTHF, 5’-methyltetrahydrofolate; NA, nicotinic acid; NAM, nicotinamide; mNAM, N1-methylnicotinamide; PA, 4-pyridoxic acid; pABG, para-aminobenzoylglutamate; PAr, PA/(PLP+PL); PL, pyridoxal; PLP, pyridoxal 5’-phosphate. (TIF) [file pone.0226069.s002.tif]

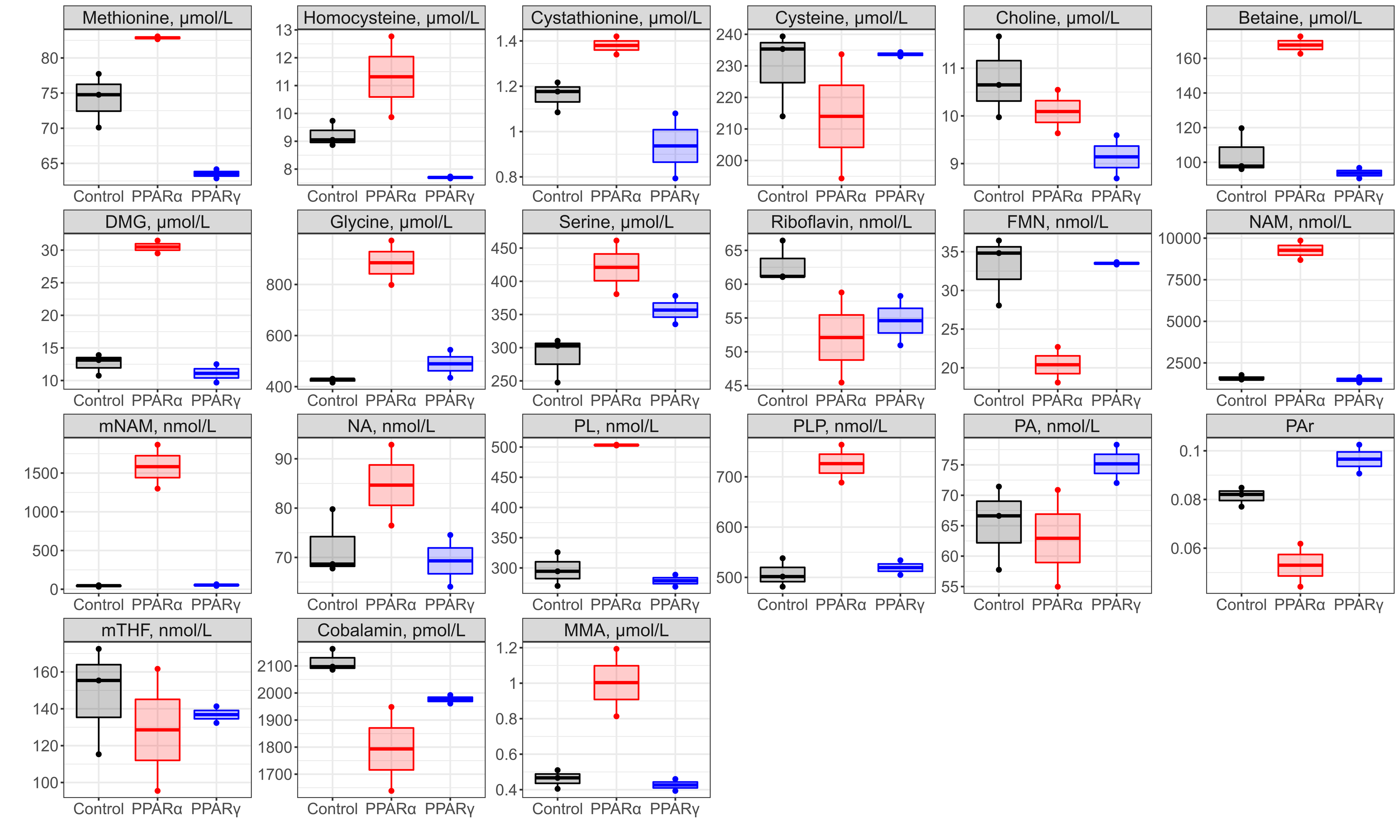

Supplement: S2 Fig — Black dots represents the control group, red dots represents the PPARα group and blue dots represents the PPARγ group. DMG, dimethylglycine; FMN, Flavin mononucleotide; MMA, methylmalonic acid; mNAM, methylnicotinamide; mTHF, 5’-methyltetrahydrofolate; NA, nicotinic acid; NAM, nicotinamide; mNAM, N1-methylnicotinamide; PA, 4-pyridoxic acid; pABG, para-aminobenzoylglutamate; PAr, PA/(PLP+PL); PL, pyridoxal; PLP, pyridoxal 5’-phosphate. (TIF) [file pone.0226069.s003.tif]
