# Supplementary material for: Short-term treatment with a peroxisome proliferator-activated receptor α agonist influences plasma one-carbon metabolites and B-vitamin status in rats
Source: PLoS One. 2019 Dec 5;14(12):e0226069. doi: 10.1371/journal.pone.0226069 (PMC6894826; doi:10.1371/journal.pone.0226069)
Supplement: Attachment — Submitted filename: Plos one-review.docx [file pone.0226069.s005.docx]

The results showed in the manuscript give some significent explain of the effect of PPARα and PPARγ on one-carbon matbolism. However, the paper is unsuitable for publication in its present form. There are some problem and suggestion as followed.

1 Energy intake should be describle more detail.
2 Although the PPARα and PPARγ mRNA expression were anaylazied ( in reference 36 ), there no difference of PPARγ mRNA level between PPARγ and control, if PPARγ was not activated in haptic and adipose tissue, how do you get the conclusion that PPARγ activatoin has rarely accosiated with one carbon matebolism? In fact, according the experiment data showed in reference 36. It seemed that PPARα mRNA expression significantly increased compared with control only in heptaic tissue, howerer, it did not seem the PPARα agonist had significant effect on PPARα expression in adipose tissue.
3 It is not approved to separate the data of PPAR expression from the data of one-carbon matebolism products. Please provide data of wester-blotting or ELISA to certify PPARα and PPARγ expression at higher level under the intervention of agonist in current study, avoiding repetition data in literature 36.
4 The describe of PPARα expression should be showed in discussion in line 91-94.
5 The analysis of genes expresssion of serine-hydroxymethyltransferase, ACMS dehydrogenase , QAPRT, methylmalonyl-CoA mutase are necessary, otherwise, the avalible evidence is not very convincing.
