# Supplementary material for: Short-term treatment with a peroxisome proliferator-activated receptor α agonist influences plasma one-carbon metabolites and B-vitamin status in rats
Source: PLoS One. 2019 Dec 5;14(12):e0226069. doi: 10.1371/journal.pone.0226069 (PMC6894826; doi:10.1371/journal.pone.0226069)
Supplement: Attachment — Submitted filename: Response to reviewers.docx [file pone.0226069.s006.docx]

Bergen, November 4^th^ 2019

Dear editor, please receive our responses to the comments raised by the reviewer (reviewer comment in bold):

**Energy intake should be described more detail.**

We have added a comment on how feed intake was estimated on line 92. Feed intake was higher in the PPARγ-group, and that group also gained slightly more total body weight as compared to the other groups.

**Although the PPARα and PPARγ mRNA expression were anaylazied ( in reference 36 ), there no difference of PPARγ mRNA level between PPARγ and control, if PPARγ was not activated in haptic and adipose tissue, how do you get the conclusion that PPARγ activatoin has rarely accosiated with one carbon matebolism? In fact, according the experiment data showed in reference 36. It seemed that PPARα mRNA expression significantly increased compared with control only in heptaic tissue, howerer, it did not seem the PPARα agonist had significant effect on PPARα expression in adipose tissue.**

Activation of PPARs does not necessarily lead to increased PPAR mRNA, so the lack of effect on the gene level should not be interpreted as an indication of lack in activation. When the PPARs are activated, the genetic expression of their target genes are induced. In our previous publication, we demonstrate large increases in the hepatic expression of several PPARα target genes (e.g. Acox1, Cd36, LPL and Hmgcs2) and adipose tissue expression of PPARγ target genes (e.g. Fatp1 and Fabp4) in the respective treatment groups. As the PPAR-activating effect of the agonists used in this experiment are well established, demonstrating this was not a goal in the current experiment. However, we are very confident that the PPARs were activated, as demonstrated by the effect on target genes.

To avoid confusion, we have replaced any reference to PPARα activation with treatment with PPARα-agonist, and PPARγ-activation with treatment with PPARγ-agonist, when discussing the present findings.

**It is not approved to separate the data of PPAR expression from the data of one-carbon matebolism products. Please provide data of wester-blotting or ELISA to** **certify PPARα and PPARγ expression at higher level under the intervention of agonist in current study, avoiding repetition data in literature 36.**

As mentioned, data on PPAR expression would not inform any inference regarding PPAR activation. Also, due to lack of biological material, we are not able to perform additional laboratory analyses from this experiment. We hope the reviewer and editor accept that we are unfortunately not able to provide this data.

We do agree with the reviewer that some indices of PPAR activation should be included in the current manuscript. However, to avoid repetition from the main publication, we have included some results in the text with reference to the original publication (reference 36) on lines 95-99, as well as mentioning this as a limitation in the discussion section.

**The describe of PPARα expression should be showed in discussion in line 91-94.**

We have added some data demonstrating PPARα and PPARγ-activation on lines 95-99, with reference to our previous publication.

**The analysis of genes expresssion of serine-hydroxymethyltransferase, ACMS dehydrogenase , QAPRT, methylmalonyl-CoA mutase are necessary, otherwise, the avalible evidence is not very convincing.**

We absolutely agree with the reviewer that this information would be very useful to aid the proposed mechanistic explanation. However, this was not included in the original protocol for the experiment, and due to lack of biological material it will unfortunately not be possible to perform additional laboratory analyses.

However, we think our data still holds value, as we show that treatment with PPAR-agonists does influence the circulating metabolites. Although we, based on our data, cannot pinpoint the exact mechanisms, the pronounced effects on these metabolites cannot be ignored. We do think that concordance with previous literature showing an effect on the gene level strengthen our results, although we were not able to measure those enzymes in this specific experiment. We have added a sentence highlighting this limitation in the discussion section.

Vegard Lysne, on behalf of the authors.
